# From the rhizosphere to systemic circulation: cross-kingdom signaling and health effects of soil microbial metabolites

**DOI:** 10.1515/biol-2025-1393

**Published:** 2026-10-27

**Authors:** Minghui Ji, Longfei Li, Lijuan Gao, Jintao Xu, Huan Liu, Yue Yao, Gang Niu, Baofeng Hao

**Affiliations:** Changli Institute of Pomology HAAFS, Qinhuangdao Hebei, 066600, China

**Keywords:** rhizosphere, systemic circulation, soil microorganisms, cross-kingdom signaling, health effects

## Abstract

Plant roots and soil microorganisms engage in continuous, complex chemical dialogues at the rhizosphere-a highly dynamic interface. The rhizosphere not only serves as a “black box” where root exudates and microbial metabolites converge but also functions as a critical site for shaping microbial community structure. Small-molecule metabolites produced by either plant roots or microbes mediate plant-microbe interactions locally and can further influence shoot physiology through systemic signaling. Similarly, in animal and human systems, gut microbes produce bioactive metabolites – such as short-chain fatty acids, indole derivatives, and secondary bile acids – that enter systemic circulation and participate in host metabolic regulation, immune modulation, and even neural function. Although the rhizosphere and gut belong to distinct ecosystems, they exhibit remarkable parallels in metabolite-mediated cross-kingdom signaling mechanisms: microbial metabolites in both systems can traverse biological barriers, enter systemic circulation, and modulate host phenotypes. Thus, from the rhizosphere to systemic circulation, soil (or gut) microbial metabolites are being redefined as pivotal mediators linking environment, microbiota, and host health. Elucidating the chemical nature, transmission pathways, and biological effects of these metabolic signals will not only advance the development of green, microbiome-based inputs for sustainable agriculture but also offer novel ecological perspectives for understanding human metabolic and neurodegenerative diseases.

## 1 Introduction

### 1.1 Ecological significance of soil microbial metabolites

Soil microbial metabolites play a central role in sustaining ecosystem functions, directly participating in organic matter dynamics and nutrient cycling processes. Agricultural sustainability depends on healthy soils and their microbial diversity. These metabolites, as direct products of soil microbial community activity, profoundly influence soil fertility and ecological balance through their composition and dynamic changes. Agricultural management practices modulate the temporal dynamics of soil microbial communities by altering soil conditions; these communities make indispensable contributions to key ecosystem functions, including organic matter transformation and nutrient cycling [1]. Therefore, understanding the ecological significance of soil microbial metabolites is critical for assessing soil health and ecosystem services.

### 1.2 Research value of the agricultural ecosystem–human health axis

As the initial environmental source of soil microbial metabolites, the health of agricultural ecosystems indirectly links to human health through impacts on soil microbial communities and their metabolic products. Studies indicate that agricultural management activities significantly alter soil conditions, thereby influencing the temporal dynamics of soil microbial communities essential to ecosystem functions. Reducing agricultural disturbance to protect soil biodiversity represents a key strategy for maintaining soil health and supporting agricultural sustainability [1]. This underscores the core value of researching the “agricultural ecosystem–human health axis”: scientifically managing agricultural soils to preserve microbial diversity and functionality may serve as a vital foundation for ensuring crop quality, promoting efficient nutrient cycling, and ultimately supporting human health. Elucidating the pathways through which soil microbial metabolites transfer from the rhizosphere environment into the food chain and affect human health provides a theoretical basis for improving public health through optimized agricultural practices.

## 2 Structural and functional characteristics of core metabolites

Soil microbial metabolites play a pivotal role in cross-kingdom signaling, with their structural and functional features directly influencing health outcomes. This section systematically elaborates on the core attributes of short-chain fatty acids, amino acid derivatives, and secondary metabolites, establishing a foundation for subsequent mechanistic investigations.

### 2.1 Chemical properties and biological activities of short-chain fatty acids

Short-chain fatty acids (SCFAs) are primarily generated by gut microbiota through the fermentation of dietary fiber. Chemically, they feature low-molecular-weight carboxylic acid structures, including acetic acid, propionic acid, and butyric acid. Functionally, SCFAs exhibit pleiotropic biological activities: they are significantly associated with neurodegenerative diseases (e.g., Parkinson’s disease), with altered SCFA levels and shifts in SCFA-producing bacterial abundance observed in patient samples [2]. SCFAs modulate immune cell function and cytokine secretion, promote vascular health (e.g., reducing blood pressure and inhibiting atherosclerosis), and enhance gut microecology by preserving intestinal mucosal barrier integrity [3], [4], [5]. Within the tumor microenvironment (TME), SCFAs act as anti-tumorigenic factors that suppress tumor progression – for instance, by regulating immune responses and mitigating inflammation [6]. Additionally, SCFAs demonstrate protective effects in disease models, such as reinforcing intestinal barrier function in affected pigs and synergistically influencing host metabolism alongside commensal bacteria (e.g., *Faecalibacterium*) [7], 8]. Further clarification is warranted that the “fatty acid” nature of SCFAs derives primarily from their carboxyl head group and short aliphatic carbon chain, typically C2–C6, rather than implying that they are major acyl constituents of triacylglycerols in human adipose tissue. In human systems, acetate, propionate and butyrate more commonly function as free acids or salts in the intestinal lumen, along the portal-hepatic axis and in the peripheral circulation, where they mediate metabolic effects and receptor-dependent signaling. Short-chain fatty acids in milk fat represent a feature of a specific lipid pool and should not be taken to indicate that general adipose triacylglycerols are dominated by SCFA acyl chains. Therefore, in this review, SCFAs are referred to as fatty acids on the basis of chemical structural classification, whereas their health effects should be understood mainly in terms of their biological distribution as microbial metabolites, local concentrations, receptor activation and epigenetic regulation [9], [10], [11], [12], [13].

### 2.2 Molecular diversity of amino acid derivatives

Amino acid derivatives display high molecular diversity, encompassing tryptophan derivatives (e.g., indoles and indole-3-propionic acid), branched-chain amino acids (BCAAs), organic acids, and carbohydrate derivatives [14], [15], [16], [17]. These metabolites arise from microbial transformation of amino acids; for example, tryptophan derivatives are microbially synthesized [14], 18], 19]. Structural variations include compounds such as p-cresol sulfate, hippuric acid, and pyrocatechol sulfate, which participate in lipid metabolism, amino acid metabolism, and hormone biosynthesis pathways [16], [20], [21], [22]. Functionally, amino acid derivatives regulate gut health – indole derivatives strengthen the intestinal barrier and suppress harmful microbes [7], 23] – while BCAAs and tryptophan derivatives modulate immune status via the gut-lung or gut-brain axes to influence physiological processes [3], 4], 18]. They are also linked to disease states: elevated levels correlate with higher frailty scores, and in metabolic syndrome, they exert effects through hepatic metabolism and inflammatory pathways [24].

### 2.3 Structure–function relationships of secondary metabolites

Secondary metabolites include secondary bile acids, polyamines, and antimicrobial peptides. Their structural features (e.g., non-peptidic or benzenoid derivatives) directly determine functional specificity [25], 26]. For instance, secondary bile acids possess hydrophobic structures and act as pro-tumorigenic factors promoting colorectal cancer (CRC) development, whereas polyamines influence tumor progression by regulating cell proliferation [6]. In microbial metabolism, these compounds participate in lipid and amino acid metabolic pathways; structural diversity (e.g., lipid-like molecules and organic acid derivatives) underpins antimicrobial properties that inhibit pathogen growth and maintain microbial community equilibrium [25], 26]. Structure–function relationships also manifest in environmental interactions: soil-derived secondary metabolites influence microbial community assembly via biosynthetic pathways and correlate with carbon-acquiring enzyme activity [20], 25]. In health contexts, secondary bile acids and polyamines serve as signaling molecules modulating inflammation and immune responses, though their effects are dose- and host-dependent [6], 18].

## 3 Metabolite transfer mechanisms in the rhizosphere-plant system

The rhizosphere serves as a dynamic interface for interactions between plant roots and soil. Its metabolite transfer mechanisms represent the core link connecting soil microbial activities with plant physiological functions. This process involves complex chemical signal exchange, selective uptake, and ecological cascade effects.

### 3.1 Metabolite exchange in microbe-plant interactions

Plant roots actively select and recruit specific functional microbial taxa from the soil by secreting targeted metabolites (e.g., root exudates), thereby establishing a highly specialized rhizosphere microbial community. These metabolites function dually as signaling molecules and nutrient substrates, governing the assembly of the rhizosphere microbiome [27], 28]. Microbes reciprocate by secreting secondary metabolites (e.g., siderophores, antibiotics) to engage in nutrient competition and establish symbiotic relationships [29]. Recent studies have identified the phenomenon of “Systemically Induced Root Exudation of Metabolites” (SIREM), wherein the rhizosphere microbiome induces systemic plant responses that reshape root exudate metabolic profiles, establishing bidirectional metabolic crosstalk [30]. This metabolic exchange is structured upon chemical gradients that form distinct microhabitats in the rhizosphere, driving spatial colonization and functional differentiation of microbes [31].

### 3.2 Key pathways of plant uptake and metabolite transformation

Plant uptake of rhizosphere metabolites exhibits high selectivity. Research confirms that specific metabolites (e.g., methionine, xanthine) significantly enhance selenium absorption efficiency through metabolite-microbe synergistic networks: these compounds promote the proliferation of selenium-enriching bacteria, increasing soil bioavailable selenium by 13.4 % and ultimately elevating plant selenium accumulation by 144 % [32]. Isoflavonoids secreted by legumes act not only as symbiotic signals for rhizobia but also modulate rhizosphere microbial community structure [33]. Benzoxazinoids (BXs) released by graminaceous crops alter the rhizosphere microenvironment, influencing the growth of subsequent crops [34]. Following uptake, plants transform exogenous metabolites via modifications such as glycosylation and methylation to reduce toxicity or enhance bioactivity – a process co-regulated by plant genotype and microbial metabolism [35].

### 3.3 Biomagnification effects in soil-crop systems

Metabolite transfer in the rhizosphere can trigger nutrient biomagnification effects. In maize intercropping systems, root-derived metabolites reshape microbial community functions (e.g., enriching phosphate-solubilizing bacteria), significantly enhancing soil phosphorus availability and increasing crop biomass by 31–92 % [36], 37]. Rhizosphere studies in rice demonstrate that zinc application suppresses ammonia volatilization and promotes nitrifier activity, elevating alkaline-hydrolyzable nitrogen content in the rhizosphere and driving a cascade amplification of nitrogen uptake efficiency [38]. Under heavy metal contamination, ferns secrete specific metabolites (e.g., organic acids) to recruit resistant microbes; these microbes alter arsenic/cadmium speciation (via redox reactions, chelation), enhancing plant accumulation capacity and forming a virtuous cycle for phytoremediation [39], 40]. This amplification effect is jointly regulated by soil physicochemical properties (e.g., aggregate structure) and microbial functional gene expression (e.g., genes encoding phosphorus-transforming enzymes) [37], 41].

## 4 Metabolite migration and transformation in the food chain

Soil microbial metabolites enter the human food chain via agricultural products, with their migration and transformation processes regulated by multiple factors. Processing of agricultural products significantly influences metabolite stability; the gastrointestinal environment determines absorption efficiency; and bioavailability within systemic circulation ultimately affects health outcomes.

### 4.1 Metabolite stability during agricultural product processing

Processing methods (e.g., thermal treatment, drying, fermentation) markedly affect the chemical stability of microbial metabolites. For instance, anthocyanin-type metabolites are prone to degradation under the influence of pH, temperature, and enzymatic hydrolysis during processing, leading to reduced bioactivity [42]. Secondary metabolites generated by microbes during processing (e.g., mycotoxins) may persist throughout the food supply chain due to thermal stability, increasing the risk of foodborne diseases [43]. Additionally, nanotechnology applied in food processing can alter metabolite release profiles; carrier materials (e.g., liposomes) enhance the stability of lipophilic metabolites [35], 40]. Food matrix components (e.g., proteins, dietary fiber) form complexes with metabolites, slowing decomposition rates during processing. Notably, acylated anthocyanins exhibit higher processing stability due to structural modifications, offering new directions for functional food development [42], 44].

### 4.2 Gastrointestinal absorption kinetics

Absorption efficiency of metabolites in the digestive tract is jointly regulated by their chemical properties and the intestinal environment. Small-molecule metabolites such as short-chain fatty acids (SCFAs) are rapidly absorbed via passive diffusion across the colonic epithelium, whereas larger molecules like polyphenols require enzymatic transformation by gut microbiota into low-molecular-weight metabolites (e.g., phenolic acids) prior to absorption [45], 46]. Lipid type critically influences absorption kinetics: medium- and long-chain triglycerides (MCT/LCT) significantly enhance intestinal uptake of hydrophobic metabolites (e.g., DDT derivatives), increasing relative bioavailability from 51.3 % to 94.6–112 % [47]. Curcumin bioavailability rises by 52 % in lipid-containing matrices, confirming that food matrices promote micellization to improve solubility of hydrophobic metabolites [48], 49]. Intestinal pathological states also markedly affect absorption: inflammatory bowel disease (IBD) models demonstrate that intestinal barrier damage increases bioavailability of perfluoroalkyl substances (PFAS) by 2.3-fold [50]. For SCFAs, lipid-matrix-driven micellization is unlikely to be the dominant explanatory mechanism. Their uptake is governed more by intestinal luminal concentration, epithelial transport and diffusion, first-pass metabolism after portal delivery to the liver, and local receptor activation thresholds. Accordingly, the manuscript’s discussion of MCT/LCT-enhanced absorption should be interpreted as applying primarily to hydrophobic metabolites and should not be simply extrapolated to SCFAs as if they were absorbed as acyl components of triacylglycerols [10], 11].

### 4.3 Bioavailability studies in systemic circulation

Bioavailability of metabolites in systemic circulation depends on their distribution, metabolism, and excretion kinetics. Although microbial metabolites (e.g., SCFAs) exhibit low oral bioavailability (∼0.2 %), their systemic health effects mediated through gut microbiota homeostasis regulation far exceed contributions from direct absorption [45], 51]. Polyphenol metabolites undergo hepatic metabolism (e.g., glucuronidation), resulting in extremely low plasma concentrations of parent compounds; metabolites (e.g., naringenin-7-glucuronide) become the predominant circulating forms, with a half-life of approximately 3 h and total bioavailability below 1 % [46], 52]. Bioavailability assessment must distinguish between “theoretical bioavailability” (parent compound) and “apparent bioavailability” (total including metabolites): flavonol metabolites transformed by gut microbiota may exhibit higher bioactivity than the parent compound [53], 54]. Carotenoid bioavailability is modulated by food matrices; lipid emulsions increase absorption rates by 35 %, while nanoemulsification technology further optimizes delivery efficiency by increasing specific surface area [55], [56], [57]. In particular, it is important to distinguish “low peripheral concentrations of parent SCFAs” from “low local bioavailability”. Butyrate is largely utilized by colonocytes, propionate is primarily transported via the portal vein for hepatic metabolism, and acetate is comparatively more likely to enter the peripheral circulation. Therefore, functional evaluation of SCFAs should be centered on tissue- and compartment-specific distribution and receptor exposure, rather than on peripheral blood concentrations alone [10], 13].

## 5 Molecular mechanisms underlying health effects

Microbial metabolites influence host health through multiple molecular pathways, with mechanisms primarily centered on three core domains: immune regulation, gut microbiota-host interactions, and intervention in metabolic syndrome.

### 5.1 Signaling pathways of immunomodulatory effects

Microbial metabolites modulate host immune responses by targeting immune cell differentiation and function. Short-chain fatty acids (SCFAs) inhibit histone deacetylase activity, promote regulatory T cell (Treg) differentiation, and enhance immune tolerance [50]. Tryptophan metabolites (e.g., indole derivatives) regulate the Th17/Treg balance and suppress pro-inflammatory cytokine production via activation of the aryl hydrocarbon receptor (AhR) [58], 59]. SCFAs and tryptophan metabolites have been demonstrated to regulate diabetes-associated immune responses through signaling pathways such as IL-17 and PI3K/AKT [60]. Additionally, microbial metabolites act as “messenger molecules,” binding to receptors (e.g., G protein-coupled receptors) on intestinal immune cells to directly modulate immune cell differentiation and function [61], 62]. Metabolites such as polyamines and polysaccharide A further regulate cytokine gene expression via epigenetic modifications (e.g., DNA methylation, histone acetylation), thereby influencing systemic immune homeostasis [63], 64].

### 5.2 Molecular basis of gut microbiota-host interactions

Microbial metabolites serve as central mediators in microbiota-host crosstalk. SCFAs (acetate, propionate, butyrate) and tryptophan metabolites enhance intestinal barrier integrity and regulate tight junction protein expression by activating FFAR2/3 receptors on intestinal epithelial cells [44], 65]. Secondary bile acids, generated from primary bile acids via microbial metabolism, modulate host lipid and glucose metabolism through the FXR/TGR5 receptor axis [66], 67]. Studies indicate that over 67 % of gut metabolites (e.g., phenyl derivatives, lipids, organic acids) are directly derived from microbial metabolism of amino acids and lipids (with classification profiles highly similar to soil microbial metabolite spectra); these molecules precisely regulate physiological functions via “metabolite–host receptor” interaction modes [55], 68], 69]. However, causal links between microbial genetic regulatory networks and metabolite biosynthesis remain unclear, representing a bottleneck in mechanistic research [70].

### 5.3 Intervention mechanisms in metabolic syndrome

Dysregulation of microbiota-derived metabolites drives metabolic syndrome through three core pathways: (1) Energy metabolism disorder: SCFAs improve insulin sensitivity by stimulating intestinal GLP-1 secretion; however, microbiota dysbiosis-induced SCFA reduction can trigger insulin resistance [44], 71]; (2) Chronic inflammation initiation: Microbiota-derived lipopolysaccharide (LPS) activates the NF-κB pathway via TLR4 receptors, inducing low-grade chronic inflammation that promotes adipose tissue inflammation and insulin resistance [72], 73]; (3) Epigenetic regulation: Butyrate upregulates PPARγ gene expression by inhibiting histone deacetylases (HDACs), thereby modulating lipid storage and oxidation [50], 74]. Mendelian randomization studies confirm a positive causal association between increased abundance of Ruminococcus and metabolic syndrome incidence (OR = 1.24, *p* < 0.001), whereas Faecalibacterium shows a negative correlation [75], 76]. Interventional studies demonstrate that alterations in gut metabolite profiles (e.g., elevated branched-chain amino acids, reduced indolepropionic acid) may serve as biomarkers for obesity and metabolic disorders [77], 78].

## 6 Methodological challenges and technological breakthroughs

### 6.1 Research paradigm shift from correlation to causal verification

Current studies predominantly rely on statistical correlation analyses, yet fail to establish causal relationships between microbial metabolites and health outcomes. For instance, statistical associations between circulating metabolites or gut microbiota ecology and health indicators (e.g., cross-sectional studies) are prone to confounding factors and cannot confirm the direct biological effects of metabolites [75]. A paradigm shift from correlation to causation requires experimental designs (e.g., interventional studies) combined with mechanistic validation.

### 6.2 Application of stable isotope tracing technology

Stable isotope labeling enables dynamic tracking of metabolite migration and transformation pathways across soil-plant-human systems. For example, labeling specific microbial metabolites (e.g., short-chain fatty acids) allows quantification of their release in the rhizosphere, plant uptake efficiency, and bioavailability within the digestive tract, revealing kinetic features of cross-system transfer [54]. This technique provides direct evidence for elucidating bioamplification effects of metabolites along the food chain. On this basis, stable isotope probing coupled with NanoSIMS can localize the true incorporation of ^13^C-, ^15^N- or ^2^H- labelled substrates at single-cell or population-specific resolution, directly revealing metabolic heterogeneity between active and inactive subpopulations within the same community. Single-cell SIP studies of the gut microbiota have demonstrated that this strategy can identify which microorganisms actually utilize specific host-derived substrates [79], [80], [81]. Unlike the mixed, community-averaged signals generated by metagenomics or bulk metabolomics, such imaging data do not infer fluxes indirectly from models but record real isotope fluxes. Mass spectrometry imaging approaches, including ToF-SIMS and 3D-OrbiSIMS, can further complement NanoSIMS by mapping the spatial distribution of lipids and small-molecule metabolites, making these combined platforms particularly suitable for flux visualization at the scale of rhizosphere microdomains and biofilms [82], 83].

### 6.3 Multi-omics integrated analysis approaches

Integrating multi-omics technologies – such as metagenomics and metabolomics – is essential for deciphering microbial metabolite functions. For instance: metagenomics identifies functional genes linked to metabolite biosynthesis (e.g., secondary metabolite pathways) within soil or gut microbial communities [46], 84]; metabolomics quantitatively profiles metabolites in rhizosphere or gut environments, pinpointing microbially associated differential metabolites (e.g., tyrosine metabolites, steroids) [54], 85]. Multi-omics correlation methods integrate microbial community structure with metabolite data to construct microbe-metabolite-host phenotype networks (e.g., gut microbiota modulation of diabetes-related signaling pathways such as IL-17, PI3K/AKT) [85], [86], [87].

### 6.4 Validation models using organoids and microfluidic systems

Organoid models simulate host tissue microenvironments to validate biological effects of microbial metabolites. For example, gut organoids elucidate mechanisms by which microbial metabolites (e.g., tryptophan derivatives) regulate intestinal epithelial barrier function and immune responses via the AhR receptor [69], 78]. Microfluidic systems reconstruct soil-rhizosphere or gut-vascular biomimetic interfaces to monitor real-time metabolite trans-barrier transport (e.g., kinetics of plant uptake of rhizosphere metabolites). These models bridge the gap between *in vitro* assays and animal models, providing cellular-level evidence for causal mechanisms [69], 84]. The extent to which these models approximate real processes should be evaluated in a tiered manner. Gut-on-a-chip systems can already maintain complex human-derived gut microbiota under controlled fluid shear, peristalsis-like mechanical forces, epithelial barrier conditions, and anaerobic/aerobic interfaces. They are therefore relatively successful for validating metabolite trans-epithelial transport, barrier-associated inflammatory responses, and microbiota-host interactions [88], 89]. Plant microfluidic platforms, such as RootChip, can also establish visualizable nutrient or metabolite gradients around living roots, enabling observation of root-tip responses and rhizosphere microdomain processes [90]. Nevertheless, these systems cannot fully reproduce soil aggregate architecture, long-term immune-neuroendocrine feedback, whole-plant source-sink relationships, or systemic regulation by diet and host metabolism. They are therefore better suited as platforms for mechanistic validation and parameterization, rather than as substitutes for field, animal, or population-level evidence.

## 7 Current controversies and theoretical challenges

Significant interspecies differences exist in dose-effect responses to soil microbial metabolites. Research indicates that microbial diversity tends to increase under moderate exposure doses but declines markedly at high doses, forming a nonlinear relationship. For instance, gut microbial communities exhibit dose-dependent responses to chemical exposures: moderate doses enhance diversity, whereas high doses reduce it [63]. Moreover, following exposure to metabolites such as bisphenol A (BPA), most metabolites in the purine metabolic pathway display a biphasic dose-response – promoted at low doses and inhibited at high doses [91]. These discrepancies may relate to host species’ metabolic capacities, microbial community structures, and detoxification mechanisms. In mouse models, pathogen colonization scales proportionally with exposure dose; however, microbial dysbiosis disrupts this dose dependency, underscoring species-specific microbial composition as a critical determinant of dose-effect variation [92], 93].

For microbially derived bioactive molecules, precise concentration dynamics should be treated as a central variable for interpreting dose-dependent effects. Rhizosphere signaling compounds often act within extremely narrow, ultra-low concentration windows. For example, strigolactones and related symbiotic signals can induce mitochondrial activation and hyphal branching in arbuscular mycorrhizal fungi at approximately 10^−9^ to 10^−8^ M, whereas concentrations above the optimal window may attenuate the expected promoting effects through receptor desensitization, metabolic feedback or altered community competition [94], 95]. Similarly, in the gut ecosystem, B-group vitamins can function both as microbial cofactors and cross-feeding molecules, while also reshaping competitive relationships within the community. SCFAs typically act at millimolar concentrations in the intestinal lumen and at lower levels in the portal and peripheral circulation, with receptor activation and HDAC inhibition showing clear compartment-dependent thresholds [13], 96], 97]. Thus, rhizosphere phytohormones, microbial vitamins and SCFAs all reflect a general homeostatic principle of low-dose signaling, an optimal activity window and high-dose feedback inhibition. Manuscript interpretation should therefore move beyond reporting presence or absence alone and should also specify concentration, exposure duration, spatial location and potential biphasic effects.

Environmental factors (e.g., temperature, soil type) and host genotype jointly regulate microbial communities and their metabolite interactions. Host genotype significantly shapes rhizosphere microbiota composition, with consistent effects observed across diverse environmental contexts [98], [99], [100]. For example, the microbial community of the sea anemone (*Nematostella vectensis*) exhibits plastic responses to environmental temperature shifts, yet response patterns are modulated by host genotype [101]. In agricultural systems, grape rootstock genotype drives microbial community reassembly via alterations in rhizosphere metabolites, a process further regulated by soil microbial chemical properties [74]. Genotype–environment interaction models further confirm that environmental factors (e.g., soil management practices) and host genetic variation collectively determine metabolite accumulation and microbial functionality [102], 103]. Additionally, interindividual variation in gut microbial responses to diet is influenced by host genetic background, highlighting the complexity of gene–environment interplay [104], [105], [106].

Microbial metabolic networks exhibit high functional redundancy; inhibition of a single pathway may trigger compensatory mechanisms, complicating health outcome predictions. For example, during host–microbe co-metabolism of xenobiotics, bidirectional interactions occur: microbial metabolites can modulate host enzyme activity, while host metabolism further transforms microbial derivatives [63], 107]. Such cascading reactions form dynamic metabolic networks wherein physiological impacts of specific metabolite loss may be compensated by alternative pathways [108]. Furthermore, the toxicity of environmental pollutants (e.g., polycyclic aromatic hydrocarbons) depends on microbe–host metabolic crosstalk, yet single-system laboratory studies inadequately capture such network complexity [109]. In dose-response investigations, metabolite disruptions often involve multiple pathways (e.g., purine/pyrimidine metabolism), while shifts in microbial community structure (e.g., enrichment of Pseudomonas) may counteract stress via metabolic remodeling, further obscuring causal attribution. Therefore, deciphering metabolic compensation necessitates integration of multi-omics approaches and organoid models to elucidate network-level regulatory cascades [67], 91], 108], 110].

## 8 Future research directions

### 8.1 Metabolite tracking technologies across ecosystems

Future research should develop high-throughput technologies to trace the dynamic pathways of soil microbial metabolites from the rhizosphere to human circulation. Integrating metagenomics, metabolomics, and stable isotope tracing techniques can elucidate the migration patterns and transformation mechanisms of metabolites within the soil–plant–food chain [111], 112]. Key breakthrough directions include: establishing multi-omics integration platforms (e.g., simultaneous metagenomic and NMR analysis) to achieve quantitative visualization of metabolic flux [113], 114]; applying machine learning algorithms to correlate environmental microbial functional genes with metabolite profiles and predict their transfer efficiency across ecosystems [111]. Additionally, optimizing enrichment and detection methods for trace metabolites in environmental samples is essential to improve the tracking accuracy of biomagnification effects in soil–crop systems [115], 116].

### 8.2 Health outcome prediction models for precision agriculture

Constructing agriculture health outcome prediction models based on microbiome functions is a core objective. By integrating soil microbial diversity indices (e.g., microbial nutrient cycling potential, abundance of pathogen-suppression genes) with crop metabolite profile data, the contribution of agricultural management practices to food nutritional quality and health effects can be quantified [112], 117]. A critical challenge lies in developing multi-scale models: at the microscale, deciphering how root exudate–microbe interaction networks influence crop metabolite synthesis [118], 119]; at the macroscale, coupling environmental variables (e.g., degree of soil degradation, pesticide residues) with human microbial exposure risks [120], 121]. Development of open-source databases (e.g., MicroAgroBiome platform) is needed to standardize agricultural soil microbiome and metabolite data, supporting cross-regional health predictions [122], 123].

### 8.3 Targeted regulation strategies for synthetic microbial communities

Designing synthetic microbial communities (SynComs) should focus on metabolite-mediated microbial recruitment mechanisms. Based on metabolites specifically secreted by plant genotypes (e.g., benzoxazinoids, coumarins), functionally modular microbial consortia can be constructed to enhance crop stress resistance and nutrient enrichment capacity [118], 124], 125]. Experimental validation should combine organoid models and microfluidic systems to simulate microbial colonization dynamics under rhizosphere metabolite gradients [126], 127]. Furthermore, metabolic network models can predict the recruitment effects of key metabolites (e.g., phenylalanine) on core microbial taxa (e.g., Streptomyces), with soil microcosm experiments optimizing SynComs field stability [128], 129]. Ultimately, metabolite-targeted regulation can enhance soil health and the medical value of agricultural products [130].

## 9 Summary and core biological insights

### 9.1 The ecological–medical bridging role of microbial metabolites

Soil microbial metabolites serve as critical mediators linking ecosystems and human health, with their mechanisms widely validated. Rhizosphere microbes produce bioactive molecules – such as short-chain fatty acids (SCFAs), amino acid derivatives, and secondary metabolites – that directly regulate plant health and nutrient uptake [102], 131]. Within the human gut, these metabolites further interact with the host immune system: SCFAs modulate Treg cell differentiation to maintain immune homeostasis, while tryptophan metabolites (e.g., indole derivatives) activate the aryl hydrocarbon receptor (AhR) pathway to sustain intestinal barrier function [132], 133]. Studies confirm that over 40 soil microbiome functions directly or indirectly contribute to the health of plants, animals, and humans [134], 135], underscoring the pivotal role of microbial metabolites at the ecology–medicine interface.

### 9.2 Reconceptualizing the medical value of sustainable agriculture

Sustainable agricultural practices significantly enhance the health benefits of agricultural products by optimizing soil microbial communities. For instance, combined application of crop straw and microbial inoculants to rehabilitate degraded soils strengthens soil nutrient cycling and promotes plant biomass accumulation, thereby improving crop nutritional value [136]. Research on biochar amendment of acidic soils demonstrates its capacity to drive microbial lipid and organic acid metabolism, enhancing soil health indicators and crop stress resilience [113]. Additionally, biosolid remediation of mining-degraded soils restored microbial community diversity by 41.7 %, with enriched taxa such as Firmicutes significantly correlated with enhanced carbon metabolism functions [137]. These findings advance agricultural management from a sole yield-oriented paradigm toward a comprehensive “health outcome” evaluation framework.

### 9.3 Paradigm innovation in the one health framework

While traditional One Health frameworks emphasize pathogen surveillance, emerging paradigms expand microbial diversity considerations from pathogens to beneficial taxa and integrate ecological and evolutionary perspectives [138], 139]. Soil serves as a cornerstone of One Health, with significant overlaps observed among soil, plant, and human microbiomes; for example, specific root metabolites can concurrently shape both rhizosphere and gut microbial compositions [121], 134]. Cross-disciplinary technological integration is pivotal: multi-omics analyses reveal soil–gut metabolite network interactions, while organoid models validate direct effects of microbial metabolites on host cells. Future efforts should establish cross-ecosystem metabolite tracking platforms to shift microbial management from disease control toward proactive ecological health regulation [140].
